# Sustainable clear aligner practice in esthetic and prosthetic dentistry: a scoping review for practical implementation

**DOI:** 10.3389/froh.2026.1854402

**Published:** 2026-07-02

**Authors:** Noha Taymour, Hams H. Abdelrahman, Dina G. Hassan, Ayman R. Khalifa, Ahmed A. Abdelaziz, Mohamed Ellabban, Mai El Zayat, Mohamed G. Hassan

**Affiliations:** 1 Department of Substitutive Dental Sciences, College of Dentistry, Imam Abdulrahman Bin Faisal University, Dammam, Saudi Arabia; 2 Department of Pediatric Dentistry and Dental Public Health, Faculty of Dentistry, Alexandria University, Alexandria, Egypt; 3 Department of Medical Environmental Studies, Faculty of Graduate Studies and Environmental Research, Ain Shams University, Cairo, Egypt; 4 Department of Preventive Dentistry, College of Dentistry, Gulf Medical University, Thumbay Dental Hospital, Ajman, United Arab Emirates; 5 Department of Orthodontics, Faculty of Dentistry, Assiut University, Assiut, Egypt; 6 Division of Bone and Mineral Diseases, Department of Medicine, School of Medicine, Washington University in St. Louis, St. Louis, MO, United States

**Keywords:** clear aligner, dental practice, esthetics, ecofriendly, sustainability

## Abstract

**Purpose:**

The purpose of this scoping review is to systematically map and synthesize the available evidence on sustainable practices in Clear Aligner Therapy (CAT), including materials, workflows, and end-of-life management with specific focus on material properties and clinical applications relevant to prosthodontic practice. It aims to systematically map the available evidence on sustainable practices in clear aligner therapy (CAT), including materials, workflows, and end-of-life management, with a specific focus on practical implementation and implications for pre-restorative and prosthodontic care.

**Material and methods:**

Following PRISMA-ScR guidelines, databases including PubMed, Scopus, Web of Science, and Google Scholar were searched. *In vitro*, observational and clinical studies on sustainability and environmental impact of CAT were included. Extracted data included study characteristics, types of environmentally sustainable practices reported, key outcomes, and identified knowledge gaps.

**Result:**

Out of 419 records, 21 studies met the inclusion criteria. The field is emerging, with the majority (57.1%) published in 2024. Study designs included reviews (52.4%) and *in-vitro* studies (23.8%). Geographically, (38.1%) of the included studies were originated from India. Key sustainability drivers were environmental impact reduction and public health/well-being (each 61.9%). Reported environmental dimensions primarily focused on material/instrument use (57.1%) and chemical use (47.6%). However, significant knowledge gaps were identified, including a lack of standardized sustainability metrics, unproven clinical feasibility of biodegradable materials and recycling protocols, and an absence of validated frameworks connecting environmental practices to prosthodontic treatment planning.

**Conclusion:**

Sustainable CAT can reduce material and chemical burdens through digital workflows, the evidence base, largely comprising recent narrative and *in-vitro* studies, lacks the clinical data necessary to validate “environmentally sustainable” protocols, life-cycle impacts, and end-of-life recycling. Because significant knowledge gaps persist regarding standardized metrics and practical feasibility, direct clinical translation remains limited. Orthodontists can best advance sustainability by optimizing digital treatment planning to minimize aligner volume and utilizing structured clinical frameworks to guide responsible, minimally invasive pre-restorative care.

**Clinical implications:**

Orthodontists should consider both material properties and environmental impact when selecting clear aligners for pre-restorative tooth positioning, occlusal therapy, or interdisciplinary treatment. The “ProSustain-P” framework provides practical guidance for implementing sustainable material practices in prosthodontic workflows, potentially reducing costs while addressing growing patient demand for environmentally responsible treatment options.

## GRAPHICAL ABSTRACT

**Figure:**
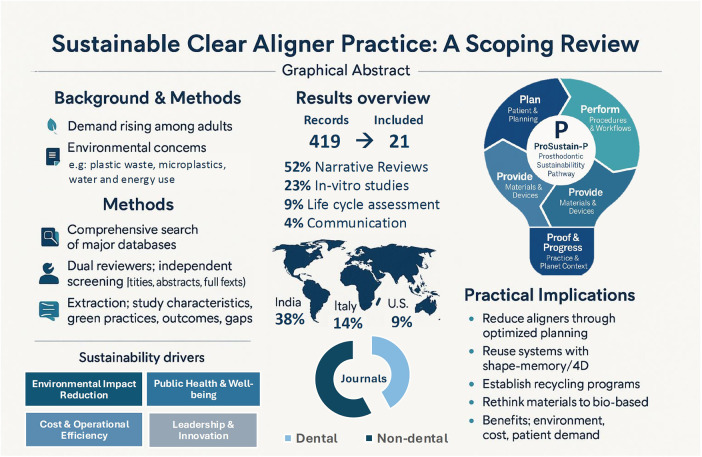

## Introduction

Clear Aligner Therapy (CAT) have rapidly transformed dental practice over the past five years, offering attractive and user-friendly alternative to traditional orthodontic braces for both orthodontic and pre-restorative prosthodontic applications (1, 2). This advancement offers aesthetic, comfortable correction of malocclusions and tooth positioning prior to definitive restorative treatment (3). These custom-fabricated resin appliances, made from transparent thermoplastic materials, reposition teeth through a series of sequential aligners making them particularly valuable in interdisciplinary prosthodontic-orthodontic treatment planning (4). Their widespread acceptance, particularly among adults seeking discreet therapeutic solutions, has fueled rapid adoption and market expansion (3, 5, 6). By 2023, more than 17 million individuals had undergone CAT worldwide (7). This growth was further accelerated during the COVID-19 pandemic due to heightened infection control protocols (8), as well as by cultural shifts emphasizing dental aesthetics (9, 10). Today, CAT industry is characterized by intense competition, continuous innovation, and direct-to-consumer marketing strategies, which collectively broaden accessibility and increase patient appeal across dental specialities (1, 11).

The treatment protocol involves sequential use of multiple aligners, typically 20–40 sets per patient, each discarded after one to two weeks, generating substantial plastic waste (12). A single treatment generates approximately 6,400 cm^2^ of thermoplastic material, and with millions treated worldwide, the cumulative environmental impact becomes significant (13). Manufacturing processes, including CAD/CAM, stereolithography, and direct 3D printing are energy-intensive and contribute to greenhouse gas emissions due to the petroleum-based nature of aligner materials (14). While clinical and commercial success is undeniable, the rapid global expansion of aligners has raised environmental concerns (15). The manufacturing process relies heavily on digital technologies and advanced materials, with aligners fabricated from petroleum-derived thermoplastic polymers including polyurethane (PU), polyethylene terephthalate glycol (PETG), and thermoplastic polyurethane (TPU) (16), chosen for transparency, flexibility, and durability, essential for effective tooth movement, patient comfort and esthetic acceptability in prosthodontic applications (4). Prosthodontics, as a specialty integrating esthetics, function, and material science, is increasingly adopting sustainable practices in response to ecological concerns (17–19). Sustainability is a key healthcare focus due to climate change, resource depletion, and environmental degradation driven by industrial and medical activities intensifies. The healthcare sector produces about 4%–5% of global greenhouse gas emissions, with dentistry contributing through energy-intensive equipment, water consumption, travel emissions, and biomedical and plastic waste. This environmental burden is prompting a shift toward “environmentally sustainable practice” in prosthodontics and restorative dentistry; however, this transition is not driven by a single motive. Sustainability drivers are defined as the primary motivating factors or enabling conditions identified in literature that facilitate or necessitate the adoption of sustainable practices in clear aligner therapy (CAT). These drivers typically encompass environmental impact reduction, public health protection, cost and operational efficiency, corporate responsibility, and technological innovation. Understanding how these sustainability drivers intersect with the unique material and clinical requirements of prosthodontics faces distinct challenges in balancing material performance with environmental responsibility (19).

Recent evidence suggests that pre-restorative clear aligner therapy can significantly enhance restorative treatment planning outcomes while minimizing the need for more invasive prosthetic preparations (2, 20–23). Simultaneously, the environmental impact of dental materials and procedures in prosthodontics has become a critical consideration for practitioners. Some aligner companies have recycling programs, enabling patients to return used aligners for processing into raw materials (24). Some manufacturers promote BPA-free, recyclable, or biodegradable plastics, though their clinical effectiveness and environmental benefits remain unproven (25). Innovations like advanced treatment planning reduce the aligners number per case, lowering plastic use (13). Direct 3D printing minimizes waste, streamlines supply chains, and improves precision and customization which are critical factors in pre-restorative applications where dimensional accuracy directly impacts subsequent prosthodontic treatment (26). Research into biodegradable aligner materials offers potential compostable alternatives, though clinical viability remains under investigation (27).

Despite promising developments, challenges persist in implementing sustainable practices. The multi-layered composition of aligners, providing rigidity and elasticity, complicates recycling efforts. Additionally, used aligners are classified as medical waste due to saliva and food contamination, hindering recycling and requiring specialized disposal protocols in dental practice. Therefore, the dental profession is urged to adopt comprehensive sustainability principles, including the reduce, reuse, recycle, and rethink (4Rs) framework to enhance environmental stewardship (13). Understanding materials, processes, and sustainability barriers in current CAT models is crucial for developing environmentally responsible alternatives.

Growing awareness of environmental responsibility in healthcare necessitates examining existing evidence and knowledge gaps in sustainable CAT (18). Although recent narrative reviews emphasize environmentally sustainable workflows in dental practice (13, 18, 19), a knowledge gap remains regarding research supporting sustainable approaches in CAT for interdisciplinary prosthodontic care. Furthermore, there is a shortage of academically approved guidelines on environmentally sustainable practices in aligners workflows and how orthodontists can address recycling, new materials development, and patient education on environmental responsibility. For this reason, this scoping review aims to systematically map the available evidence on sustainable practices in clear aligner therapy (CAT), including materials, workflows, and end-of-life management, with a specific focus on practical implementation and implications for pre-restorative and prosthodontic care. By delineating these current challenges and knowledge gaps, this review lays the necessary groundwork for developing evidence-based, specialty-specific sustainability frameworks.

## Material and methods

### Study design

This study followed a scoping review design to systematically identify, synthesize, and map the available evidence on sustainable and environmentally responsible practices in CAT. The methodology was guided by the PRISMA-ScR (Preferred Reporting Items for Systematic Reviews and Meta-Analyses extension for Scoping Reviews).

### Eligibility criteria

To address the study's PICO question in the context of clear aligner therapy (CAT), what is the available evidence regarding the impact of sustainable practices across the product lifecycle on environmental indicators, resource consumption, and operational feasibility compared to conventional practices among all involved stakeholders?, we included peer-reviewed original research (clinical, *in vitro*/*in vivo*, LCA, modeling) and evidence syntheses (systematic reviews, meta-analyses, narrative and scoping reviews) published in English, with no time restrictions. Exclusions were studies unrelated to clear aligners sustainability, non-English publications, and records lacking full text. Using the PICO framework to structure the eligibility criteria, the Population (P) included all stakeholders involved in CAT (patients, clinicians/clinics, manufacturers, and the supply chain) across any clinical setting and geographic location. The Intervention (I) was any sustainable practice linked to CAT throughout the product life cycle (e.g., material innovation, waste reduction/recycling/take-back programs, energy or water efficiency, safer chemical profiles, eco-conscious packaging/logistics, and education or policy initiatives). The Comparator (C) included conventional or non-sustainable practices, alternative workflows/appliances, or none where studies were purely descriptive (e.g., LCA or modeling). Outcomes (O) required at least one measure related to environmental impact (e.g., life-cycle indicators, emissions, microplastics/toxicity), resource use (materials, energy, water), or economic/operational implications (costs, efficiency, feasibility/adoption).

### Search strategy

A comprehensive electronic search was conducted using PubMed, Web of Science, and Google Scholar. Search terms were refined iteratively and supplemented by manual screening reference lists from relevant articles.

The PubMed search strategy included MeSH terms and keywords:

(“Clear Aligners”[Mesh] OR “Aligners” OR “Invisalign” OR “Thermoplastic aligners” OR “Plastisc” OR “Microplastic” OR “Removable orthodontic appliances”) AND (“Sustainability”[Mesh] OR “Green Dentistry” OR “Green Orthodontics” OR “Eco-friendly” OR “Sustainable Materials” OR “Carbon Footprint” OR “Waste Management” OR “Recycling” OR “Biodegradable” OR “Plastic” OR “Microplastic” OR “Environmental Impact”) AND (“Orthodontics”[Mesh] OR “Dental Practice” OR “Dentistry”)

The Web of Science query was:

TS=(“clear aligners” OR “Invisalign” OR “thermoplastic aligners” OR “removable orthodontic appliances”) AND TS=(“sustainability” OR “green dentistry” OR “eco-friendly” OR “carbon footprint” OR “waste management” OR “recycling” OR “biodegradable” OR “environmental impact” OR “sustainable materials” OR “life cycle assessment”) AND TS=(“orthodontics” OR “dental practice” OR “dentistry” OR “dental materials”)

The Google Scholar query was:

“Clear Aligners” OR “Aligners” OR “Invisalign” OR “Thermoplastic Aligners” OR “Plastic” OR “Microplastic” OR “Removable Orthodontic Appliances” AND “Sustainability” OR “Green Dentistry” OR “Green Orthodontics” OR “Eco-friendly” OR “Sustainable Materials” OR “Carbon Footprint” OR “Waste Management” OR “Recycling” OR “Biodegradable” OR “Environmental Impact” AND “Orthodontics” OR “Dental Practice” OR “Dentistry”

### Study selection

Two reviewers (MGH and H.H) independently screened all titles and abstracts. To ensure consistency, reviewers independently piloted the screening process on 10 randomly selected studies. The inter-examiner reliability demonstrated strong agreement, with a Cohen's kappa score of 0.82. Full texts were retrieved for potentially eligible studies and assessed against the predefined inclusion criteria.

### Data extraction

Data were extracted using a standardized form developed specifically to characterize the literature landscape (Objective 1): We extracted study demographic data, including author(s), year of publication, country of origin, journal discipline, study design and publication type. To map environmental dimensions and interventions (Objective 2): We extracted precise details regarding the phase of the aligner lifecycle addressed. Specific interventions extracted included the type of “environmentally sustainable” practice applied. To identify sustainability drivers and reported outcomes (Objective 3): We extracted the stated motivators for sustainable practices (e.g., environmental impact reduction, public health protection, cost/operational efficiency, corporate responsibility). Outcome data were categorized into environmental indicators, resource consumption and operational/clinical implications. To isolate knowledge gaps for framework development (Objective 4): We extracted the limitations of current sustainable practices, barriers to recycling, unproven clinical viability of novel materials, and the absence of standardized clinical guidelines.

Extraction was performed independently by two reviewers (MGH and H.H) to minimize bias and error. Inter-examiner reliability, assessed on 15% of the included studies, yielded a kappa score of 0.87. Disagreements were resolved through discussion or, when necessary, consultation with a third reviewer to ensure consistency and accuracy.

### Data synthesis

A thematic analysis approach was adopted to organize and synthesize the included studies into four main sustainability domains in orthodontics: Material Reuse, Water Reduction, Energy Consumption, and Chemical Use & Environmental Impact, each comprising multiple sub-themes covering material innovation, life cycle assessment, waste management, clinical sustainability strategies, and patient/staff education. By synthesizing the specific barriers and clinical feasibility data coded within these domains, we were able to systematically identify where existing conceptual models (such as the 4Rs) failed to address prosthodontic realities, which directly informed the conceptualization and structure of the proposed ProSustain-P framework presented in the Discussion.

### Statistical analysis

All descriptive statistical analyses were conducted using Microsoft Excel and SPSS version 23 for Windows (IBM, Armonk, NY, USA). Frequencies and percentages were calculated to determine the distribution of studies across countries, year of publication, study design, journal sources, and sustainability themes.

## Results

### General characteristics

**Figure 1:**
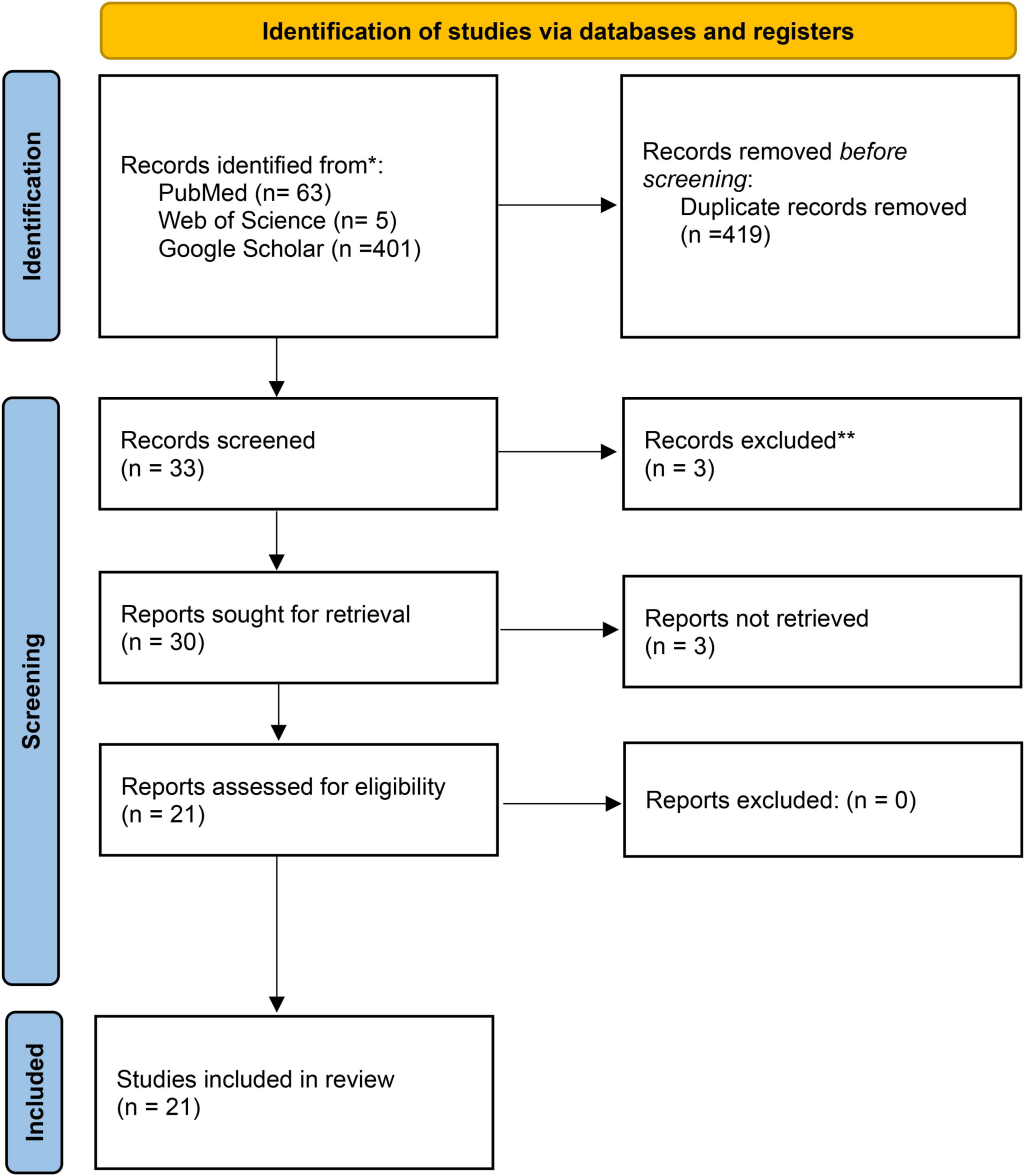
PRISMA Flow chart.

A total of 21 studies met the inclusion criteria for this scoping review, selected from an initial pool of 419 retrieved articles. After screening and assessment, 33 studies underwent detailed review, resulting in the exclusion of 9 studies due to ineligibility and 3 due to inaccessibility (Figure 1). A comprehensive synthesis of the key findings from the included studies is presented in Table 1.

**Table 1:** Characteristics and key findings of the included studies.

Author	Year	Study Design	Aim and Methods	Key Findings	Conclusion
Macrì et al. (13)	2024	*In vitro* & *in vivo*	Sustainability strategies in aligner manufacturing/use	Promotes 4Rs (Reduce, Reuse, Recycle, Rethink), patient/clinician education	Systemic changes are needed for sustainability.
Mani et al. (52)	2013	Review	Summarize microplastics in orthodontics and potential alternatives	Clear aligners and other devices release microplastics with physical/chemical/biological effects	Call for biodegradable alternatives and prevention.
Kilaru K. (53)	2021	Conference paper	Auditing the innovation of 3D printed orthodontic aligners	3D manufacturing offers benefits over thermoforming but requires custom programming	Dentistry is entering an additive manufacturing era.
Peter et al. (54)	2022	Review	Investigating the environmental impact of clear aligners	Stakeholders, including manufacturers, clinicians, and researchers, should consider life-cycle impact, waste reduction measures, and sustainable material alternatives.	Promote sustainable aligner use, materials, disposal, and treatment efficiency.
Gupta et al. (55)	2022	Review	Guidelines for aligner disposal to reduce biohazards	Disposal/recycling awareness needed to protect environment	Promote systematic disposal/recycling.
Caelli et al. (56)	2023	Comparative LCA	Compare thermoforming vs. 3D printing aligners via LCA	3D printing offers reduced environmental impacts	3D printing is more sustainable.
Tan et al. (57)	2024	Comparative LCA	Compare sustainability of Hawley vs. Essix retainers	Essix has higher environmental impact over its lifespan	Hawley retainers are more sustainable.
Raj et al. (58)	2024	Cross-sectional survey	Assess awareness of aligner disposal among dental professionals	Need for better awareness and instructions on proper disposal	Highlight the importance of disposal education.
Leach E. (59)	2024	*In vitro* (Thesis)	Determine degradation rate among Aligner brands	Aligner materials degrade very slowly outdoors—often taking 25–39 years to fully break down	a significant environmental burden from aligner disposal.
Lecocq et al. (60)	2024	Review	Review of the aligner composition, recycling, and properties	Plastic printing is not equivalent to metal wire performance	Mechanical limitations remain.
Panayi et al. (61)	2024	Review	Analyze microplastic contamination and health risks from aligners	Aligners release microplastics and may harm multiple body systems	Aligners carry serious environmental/health risks.
Akhtar et al. (62)	2024	Cross-sectional	Quantify microplastics in dental settings using dust samples	Higher microplastic exposure in teaching hospitals; PET dominant	Health risks identified, especially for dental professionals.
Veseli et al. (63)	2024	Review	Environmental impacts of aligners and carbon emission solutions	Non-biodegradable aligners persist; recycling/education needed	Support hybrid treatment and patient recycling efforts.
Ong et al. (64)	2024	Review	Scope of plastic waste and sustainability in oral healthcare	Review dental polymers, recycling limits, bioplastic potential	Urgent need for circular economy in dental materials.
Palmieri et al. (65)	2024	*In vitro*	Test mechanical/chemical stability of ClearX aligners	ClearX is reusable, stable after reshaping	4D-printed aligners reduce waste.
Choi et al. (66)	2024	*In vitro*/*in vivo*	Develop silk fibroin aligner (MSB)	High performance, antimicrobial, biocompatible, recyclable	MSB bioplastic is a promising sustainable alternative.
Peter et al. (67)	2024	*In vitro*	Analyze emissions from aligner incineration	Identified toxic/carcinogenic smoke byproducts	Biodegradable materials and proper disposal needed.
Shrivastava et al. (68)	2025	Literature review	Review of environmental sustainability measures in orthodontics	Evidence supports eco-conscious practices but emphasizes need for more *in vivo* research	Toward sustainable orthodontics with innovative approaches.
Camcı et al. (69)	2025	Review	Assess aligners’ environmental impact and propose solutions	Need government/manufacturer action to avoid increasing plastic burden	Policy changes required.
Manochehri et al. (70)	2025	Poster	Practical strategies for sustainable orthodontics	Reuse materials, improve digital workflows, energy/waste reduction	Many in-office strategies can cut environmental impact.
Gandhi et al. (71)	2023	Review article	Review disposal methods and environmental hazards of aligners	Highlight benefits/drawbacks and promote environmentally sustainable disposal	Emphasize sustainable waste management.

### Study characteristics

**Figure 2:**
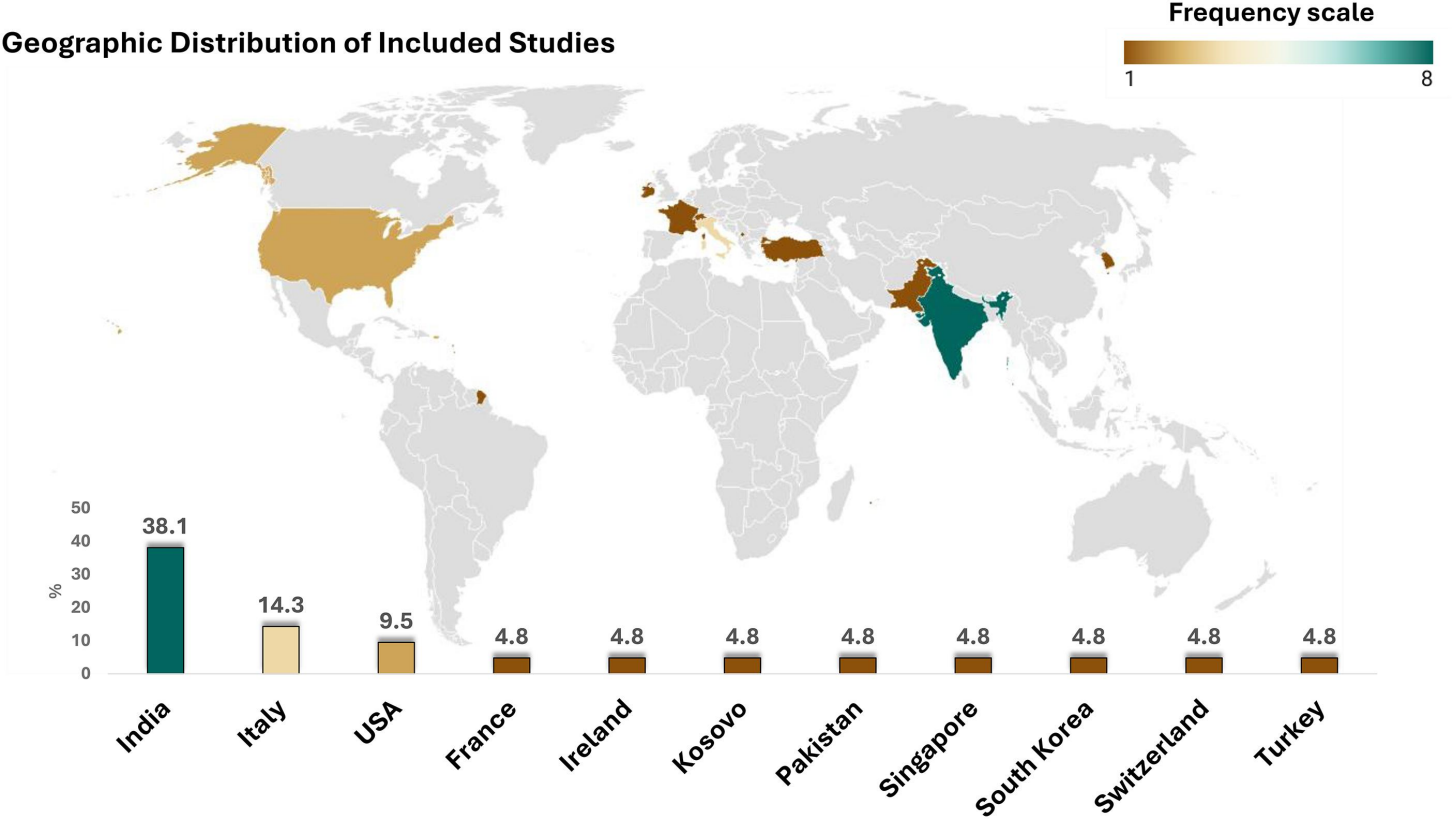
Geographic distribution of the included studies.

Geographically, the research contributions were notably diverse. India led with 8 studies (38.1%), followed by Italy with 3 studies (14.3%), and the United States contributed 2 studies (9.5%). Other countries individually contributed one study each (4.8%), demonstrating global yet concentrated research activity (Figure 2).

**Figure 3:**
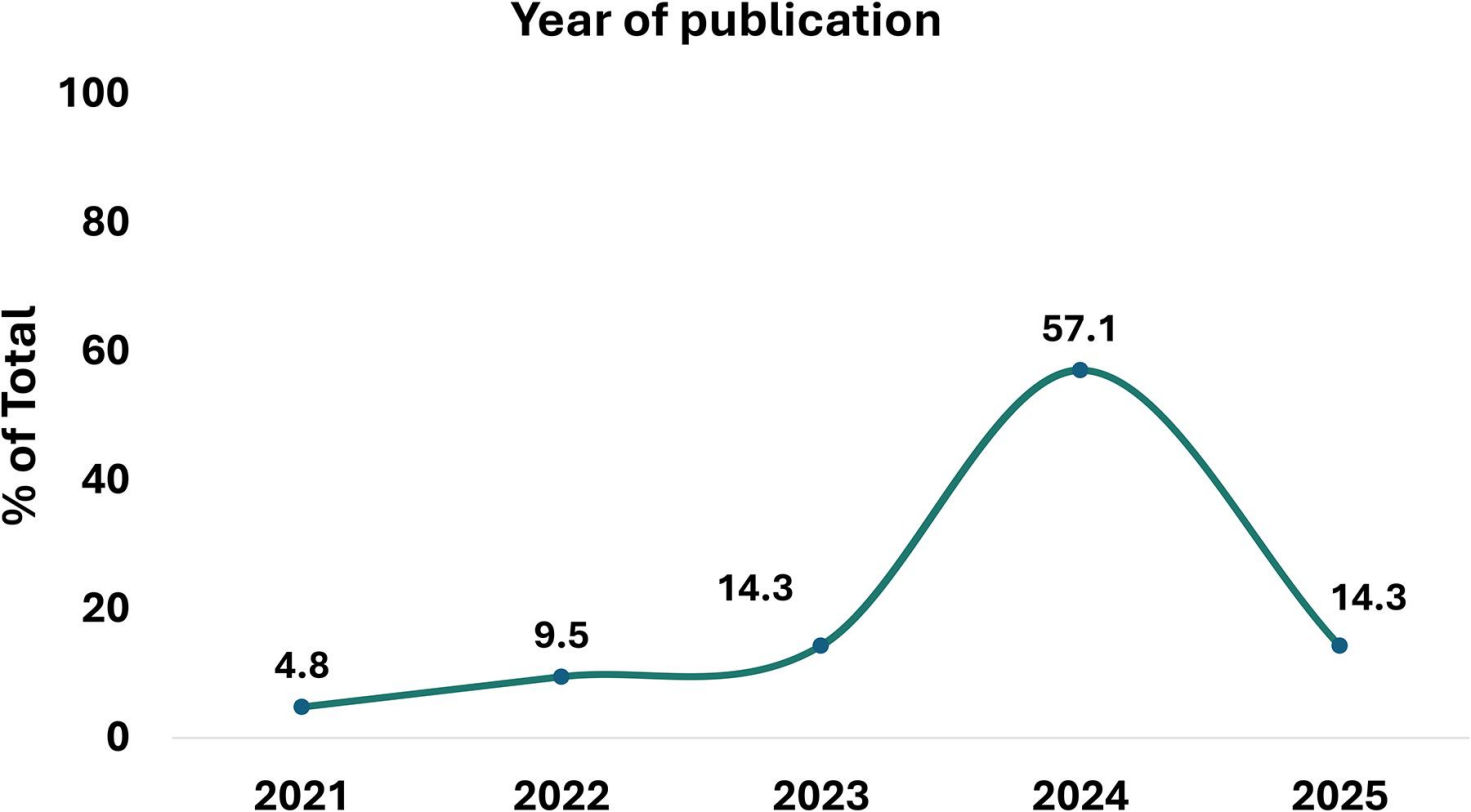
Studies distribution according to year of publication.

The publication timeline indicated a marked recent surge in interest, with the majority of studies (57.1%, 12 studies) published in 2024. Publications from 2023 to 2025 each accounted for 14.3% (3 studies), while 2022 contributed 9.5% (2 studies), and only 4.8% (1 study) appeared in 2021 (Figure 3).

Included studies represented diverse methodological approaches, with reviews constituting over half (52.4%, 11 studies), indicative of the field's developmental stage and the necessity for synthesis of existing knowledge. *In vitro* studies accounted for 23.8% (5 studies), while Life Cycle Assessment (LCA) and cross-sectional analyses each contributed 9.5% (2 studies), and a single communication piece represented 4.8% of the total studies.

The included articles appeared across various journals, both orthodontic-specific and interdisciplinary. Orthodontic journals comprised 9 studies (around 42%) across 7 journals, with the American Journal of Orthodontics and Dentofacial Orthopedics featuring most prominently (14.3%, 3 studies). Other journals each contributed a single study (4.8% each). Overall, more than half of the publishing journals were indexed in Clarivate (57.9%, 11 journals) and PubMed (52.6%, 10 journals).

### Analysis of sustainability key drivers in clear aligner practice

Analysis of sustainability drivers showed five core themes (Table 2). Environmental impact reduction and public health and well-being were most frequently reported, each addressed by 13 studies (61.9%). Studies under these themes emphasized strategies to lower carbon emissions, reduce plastic waste, mitigate toxic exposures, and safeguard clinician and patient health from environmental hazards such as microplastics. Cost savings and operational efficiency emerged as motivating factors in 10 studies (47.6%), recognizing that sustainable practices can streamline operations, decrease costs, and minimize waste. Corporate social responsibility and ethical imperatives, along with leadership and innovation in healthcare, were each highlighted in 9 studies (42.9%), emphasizing the ethical obligations and innovative opportunities in sustainable orthodontic practices.

**Table 2:** Key drivers of sustainable aims and environmental aspects addressed by studies included.

Items	Total = 21 studies
Key drivers of sustainable aims	
Environmental impact reduction^13, 52, 56, 57, 62, 63, 64, 65, 66, 67, 68, 69,70^	13 (61.99%)
Public health and well-being^13, 52, 58, 61, 62, 63, 64, 65, 66,67, 68, 70, 71^	13 (61.90%)
Cost savings and operational efficiency^13, 56, 57, 63, 64, 65, 66, 67, 68, 70^	10 (47.86%)
Corporate social responsibility and ethical imperatives^13, 58, 61, 62, 63, 64, 65, 66, 67^	9 (42.86%)
Leadership and innovation in healthcare^13, 53, 60, 61, 63, 64, 65, 66, 67^	9 (42.86%)
Environmental aspects	
Instrument (material use)^13, 52, 53, 55, 58, 63, 64, 65, 66, 68, 70, 71^	12 (57.14%
Water reduction^56, 57, 65, 70^	4 (19.05%)
Energy consumption^13, 52, 54, 56, 65, 67, 70^	7 (33.33%)
Chemical use^13, 52, 56, 61, 64, 65, 66, 67, 69, 70^	10 (47.62%)

### Analysis of environmental dimensions in CAT

Specific environmental dimensions addressed in the reviewed literature (Table 2) included instrument and material use, reported by 12 studies (57.1%). These investigations advocated bio-based, biodegradable materials (PLA, PHAs, silk fibroin) and innovative recycling technologies (glycolysis, aminolysis). Chemical use was critically discussed by 10 studies (47.6%), with recommendations to adopt safer materials, reduce toxic additives, and implement effective chemical recycling methods. Energy consumption was featured in 7 studies (33.3%), highlighting the benefits of transitioning from energy-intensive thermoforming to efficient 3D/4D printing and utilizing energy-efficient clinical equipment. Water reduction, while least frequently addressed (19.1%, 4 studies), underscored the potential of digital fabrication methods and efficient dental equipment to significantly lower water usage.

### Thematic analysis

A thematic synthesis of the included studies identified and systematically extracted, categorized, and synthesized the existing recommendations and proposed strategies mentioned the included studies. By coding the qualitative data against the four *a priori* environmental domains established in the methods (material/instrument use, water reduction, energy consumption, and chemical use), the synthesis mapped how the current literature conceptualizes sustainable workflows (Table 3). Within instrument reuse, the principles of “4Rs” (Reduce, Reuse, Recycle, Rethink) were emphasized, recommending advanced technologies like shape-memory polymers and 4D printing for multiple-stage aligner reuse, clear disposal procedures, incentives for sustainable clinical behaviors, and increased public and professional awareness.

**Table 3:** Guidelines and key recommendations according to sustainability environmental aspects addressed by the included studies.

Main theme	Sub-theme	Guideline description	Key recommendations
1. Material Reuse	1.1 Enabling Direct Aligner Reuse through Advanced Technologies^13, 65^	Use of 4D printing and shape-memory polymers to reshape and reuse aligners across multiple treatment stages.	Adopt ClearX or similar shape-memory aligners to reduce plastic use per patient by reusing a single aligner after thermal activation.
	1.2 Fostering Comprehensive Material Recycling and Upcycling^52, 55, 58, 63, 64, 71^	Chemical and mechanical recycling processes that recover and reuse materials, promoting circular economy principles.	Utilize glycolysis, aminolysis, and mechanical recycling; develop collection systems and manufacturer-clinician partnerships; implement zip-lock/red-bag disposal; encourage upcycling of polymers.
	1.3 Innovating with Sustainable and Bio-based Materials^52, 64, 66^	Development of biodegradable and recyclable alternatives to conventional plastics.	Adopt materials such as silk fibroin, PLA, and PHAs for aligners; use antimicrobial and recyclable polymers to minimize chemical inputs and waste.
	1.4 Developing Policy, Education & Collaborative Frameworks^58, 71^	Emphasizing the role of dental bodies and institutions in shaping sustainable practices.	Establish national/regional disposal protocols; offer incentives for recycling; educate manufacturers and clinicians; promote collaborations between dental councils, manufacturers, and clinics.
	1.5 Embracing the 4Rs Framework^13, 68^	Adopting the Reduce, Reuse, Recycle, Rethink philosophy across orthodontic workflows.	Incorporate sustainability thinking in all aligner-related decisions; reduce waste through reuse and rethink treatment models that require fewer devices and lower emissions.
2. Water Reduction	2.1 Water-Efficient Manufacturing^56, 65^	Reducing water use in aligner production by replacing thermoforming with digital fabrication.	Use Direct Light Processing (DLP) 3D printing to minimize post-processing water usage and eliminate model washing.
	2.2 Implementing Water-Saving Clinical Practices^71^	Encouraging water-saving behaviors and technologies in clinics.	Use water-efficient dental chairs and digital impressions; reduce water-dependent activities.
	2.3 Indirect Water Savings from Reuse^65^	Lower aligner production volume indirectly leads to reduced water consumption.	Fewer aligners required per patient (via reuse) reduces total water used across the product lifecycle.
	2.4 Considering Appliance Choice^57^	Selecting appliance types with lower environmental water impact.	Choose Hawley retainers over Essix due to superior environmental profile.
3. Energy Consumption	3.1 Enhancing Manufacturing Efficiency^52, 56, 67^	Lowering energy use in aligner production through technological upgrades.	Use direct 3D printing instead of thermoforming; avoid overproduction; implement energy-efficient equipment and batch planning.
	3.2 Optimizing Clinical Energy Practices^70^	Promote low-energy infrastructure and clinic operations.	Use motion sensors, LED lighting, digital workflows, and energy-saving HVAC systems; power down idle machines.
	3.3 Reducing Lifecycle Energy Burden^54, 65^	Designing treatment strategies to minimize energy across product use and disposal.	Reduce aligner change frequency; reuse aligners using 4D tech; reduce packaging and transportation needs.
	3.4 Strategic Planning & Ordering^13, 67^	Reducing energy waste through efficient supply chain and clinical protocols.	Order aligners in phases, not all at once; plan treatments to avoid unused devices.
	3.5 Energy Conservation under the 4Rs^13^	Aligning energy reduction with broader environmental sustainability goals.	Integrate energy-saving strategies into holistic sustainable orthodontic frameworks.
4. Chemical Use & Environmental Impact	4.1 Shifting to Safer & Biocompatible Materials^13, 52, 64, 66, 69^	Reducing chemical toxicity by selecting safer, biodegradable, and recyclable aligner materials.	Use MSB, PLA, and PHAs; avoid traditional petroleum-based plastics; select materials with minimal environmental leaching.
	4.2 Minimizing Chemical Leaching & Toxicity^56, 65^	Improve safety in production and patient exposure.	Post-curing and UV treatment for 3D printed aligners; avoid additives that release microplastics or toxins.
	4.3 Advancing Chemical Recycling Techniques^64^	Chemical recycling to regenerate raw polymers from used aligners.	Adopt glycolysis, aminolysis, and other recovery methods to close the material loop.
	4.4 Reducing Reliance on External Chemicals^66^	Using materials that don't require extra chemical agents.	Use antimicrobial materials (e.g., MSB) that negate the need for chemical disinfectants or coatings.
	4.5 Establishing Waste Protocols and Education^61, 67, 69^	Creating structured systems for chemical safety and disposal.	Follow biomedical waste protocols; educate patients via booklets; involve manufacturers in safe disposal practices.
	4.6 Reducing Chemical Input through Process Design^13, 56, 65^	Indirectly reduce chemical use by changing manufacturing processes.	Reduce number of materials; use 3D printing; stage production to avoid waste; promote reshaping instead of discarding.

Recommendations on water use emphasized direct aligner 3D printing to reduce water consumption significantly compared to traditional production processes, alongside the adoption of water-saving dental equipment. Energy conservation guidelines highlighted the environmental advantages of 3D/4D printing technologies, optimized tray production schedules, and energy-efficient clinical practices such as LED lighting and efficient HVAC systems.

Finally, chemical use guidelines prioritized the replacement of traditional petrochemical-based materials with biocompatible, biodegradablealternatives such as polylactic acid (PLA), polyhydroxyalkanoates (PHAs), and silk fibroin composites. However, a critical evaluation of these recommendations reveals a significant gap between theoretical background and clinical reality. Currently, none of these biodegradable materials can be considered fully “fit for purpose” for clear aligner therapy (CAT). The dynamic intraoral environment, characterized by continuous thermal fluctuations, complex masticatory loading, and hydration, compromises the mechanical properties, force delivery, and fatigue resistance of these polymers might rendering them unreliable for predictable tooth movement. Furthermore, while these materials are theoretically biodegradable in industrial facilities, classifying used aligners as contaminated biohazardous medical waste severely complicates any real-world recycling or composting pathways. Additionally, the establishment of supportive policies from industry and governmental agencies was recommended to advance the development and adoption of environmentally friendly dental polymers and safe chemical management practices.

## Discussion

This scoping review evaluates evidence on the sustainability of CAT, a subject of growing concern for dental practice. Most studies consisted of narrative reviews and theoretical papers, with few standardize measures of sustainability or comprehensive real-world studies, thus, the hypothesis that there is comprehensive, evidence—based guidance for implementing sustainable CAT in esthetic practice was not supported. Our findings reveal a significant tension between the remarkable clinical and commercial success of CAT and its inherent environmental footprint. It highlights the necessity of making sustainability a core component of clinical dental practice and urges for assessment of materials, manufacturing processes, and waste management protocols associated with clear aligners in adjunctive prosthetic therapy (18).

Previously, several studies prioritized clinical outcomes and patient experience over sustainability imperatives (28–30). More than 100 studies focus exclusively on CAT efficacy, and patient experience (31, 32). As a result, the environmental impact of CAT remained largely unexplored until recently. Our findings show a positive change, aligning with broader discussions about sustainability in healthcare. Out of 419 identified articles, only 21 met the inclusion criteria, highlighting sustainable CAT as an emerging research domain. Despite escalating concerns over dental waste, this area remains underexplored in literature. This mirrors patterns in broader healthcare; Martín-Peláez et al. (33) found a 5.6% inclusion rate for sustainable practices in general dentistry (33). These findings show that, although research on sustainability is expanding, it still represents only a small share of academic output. The environmental burden of single-use plastics, chemical waste, and energy consumption in CAT is now acknowledged as a driver for environmentally sustainable practices (13). This shift is timely, given variation in how sustainability is conceptualized and measured in dental research.

The analysis of study characteristics revealed patterns across geographical distribution, publication timelines, methodological approaches, and journal affiliations. With 38.1% of the reviewed literature, India was the largest contributor, followed by Italy and the United States, with each other country contributing one study. India's significant contribution to the published literature on sustainable CAT suggests a potential reorientation of research priorities, possibly driven by localized environmental challenges. In contrast, the limited contributions from established clear aligner markets, such as North America and Europe, point to a gap between market dominance and engagement in sustainability research (34). This finding aligns with contemporary scholarly discourse emphasizing the increasing involvement of developing economies in research situated at the nexus of healthcare and ecological concerns (35). Analysis of the publication timeline indicates a substantial rise in sustainability-focused clear aligners research, with over half (57.1%) of the identified studies appearing in 2024. Earlier years showed far lower output: 2023 and 2025 each at 14.3%, 2022 at 9.5%, and 2021 at only 4.8%. This acceleration in research output corresponds with heightened global awareness of environmental footprints, particularly amplified by the COVID-19 pandemic's emphasis on medical waste management (36). Furthermore, the increasing stringency of regulations pertaining to plastics and carbon emissions likely serves as an additional impetus for advancements in this research domain (37). Chua MT et al. (38) and Nunes AR et al. (39) similarly observed an exponential growth in sustainable healthcare publications after 2019, attributing this trend to evolving climate policies and broader global consciousness. In the context of academic publishing, both orthodontic and interdisciplinary journals exhibit a significant presence, with 42% of the literature appearing in orthodontic journals and over half indexed in major academic databases; however, esthetic dentistry journals are absent despite the expanded everyday use of CAT in esthetic dental practice. This pattern suggests that although sustainability is increasingly recognized as a fundamental aspect of healthcare quality, esthetic dentistry specialists may not yet be fully engaged with, or supported by, specialty-specific evidence and guidance on sustainable CAT.

### Key themes and drivers of sustainability

**Figure 4:**
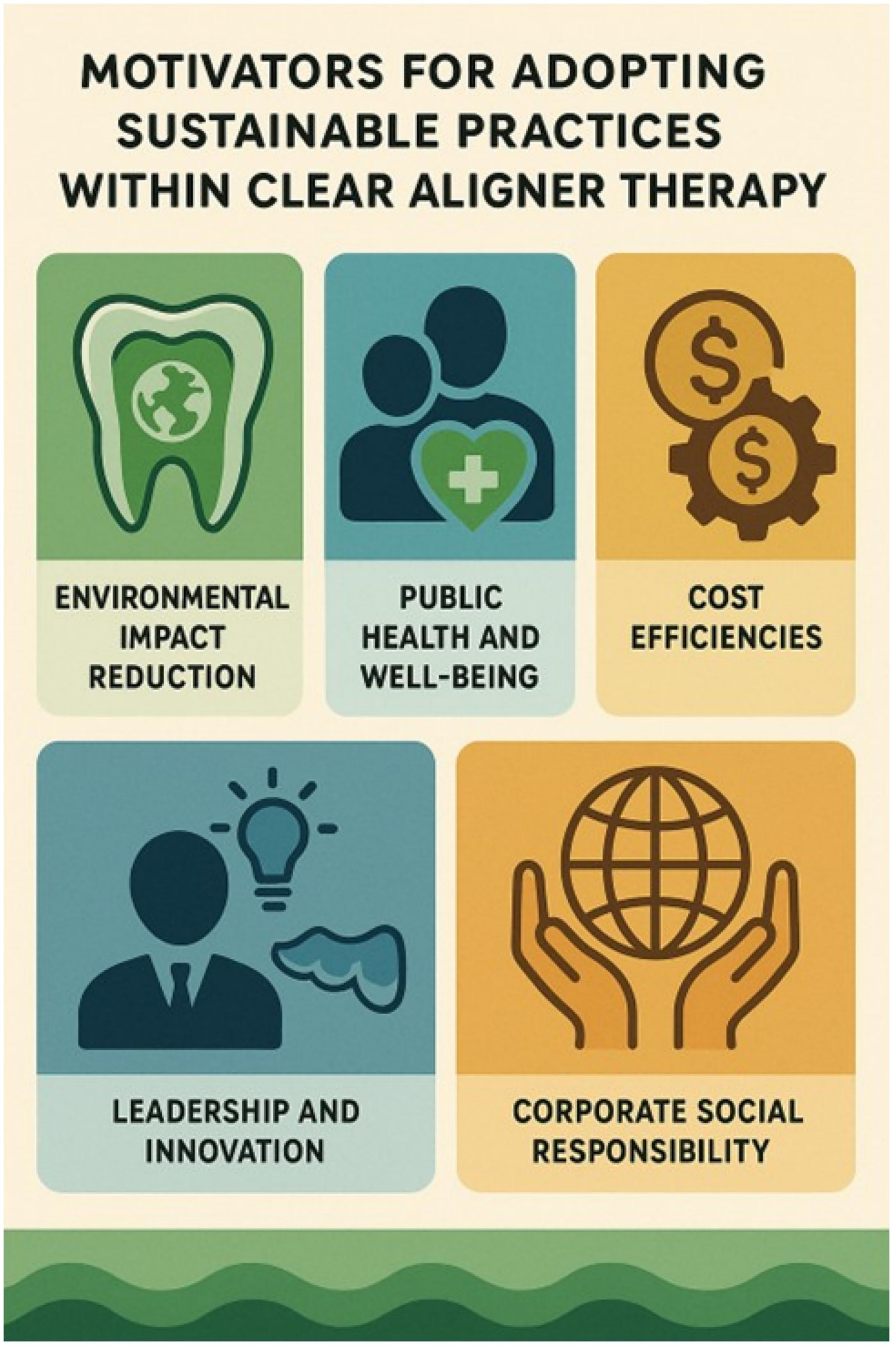
Motivators for sustainable practices adoption within clear aligner therapy.

This review systematically identified five main themes that serve as primary motivators for the adoption of sustainable practices within CAT: *environmental impact reduction, public health and well-being, cost efficiencies, leadership and innovation, and corporate social responsibility* (Figure 4). These themes demonstrate considerable congruence with the established “triple bottom line” framework, which holistically integrates environmental, social, and economic dimensions (40). Environmental impact reduction and the promotion of public health and well-being were concurrently reported as significant motivators in 61.9% of the examined studies. This prevalence underscores an escalating recognition of CAT's ecological footprint, largely attributable to the considerable plastic waste generated by 20–30 sequential aligners per treatment and the energy-intensive nature of 3D printing manufacturing processes (13). Furthermore, emergent health concerns regarding microplastic exposure and chemical leaching from plastic materials, particularly in light of recent findings detecting microplastics in human tissues, also serve as a critical impetus for research in this domain (41). Notably, cost savings and operational efficiency were emphasized in 47.6% of studies, thereby challenging the conventional assumption that sustainability inherently necessitates increased expenditure. The implementation of waste reduction strategies and digital workflows demonstrably yields both economic and environmental advantages (42). Finally, leadership, innovation, and corporate social responsibility were prominent in over 40% of the investigations, signaling an evolving professional awareness and the strategic benefits associated with sustainable innovation.

### Environmental dimensions

This review identified four key environmental dimensions relevant to sustainable CAT material/instrument use (57.1%), chemical use (47.6%), energy consumption (33.3%), and water reduction (19.1%). Material use emerged as the most frequently addressed domain, reflecting the inherently material-intensive nature of CAT. A substantial proportion of the literature advocated for a transition toward bio-based and biodegradable polymers such as polylactic acid (PLA), polyhydroxyalkanoates (PHAs) to reduce environmental persistence. However, critical appraisal exposes a disconnect between these theoretical claims and real-world reality. Clinically, these alternative materials currently lack the sustained force delivery and intraoral fatigue resistance required for predictable tooth movement. Environmentally, their proposed benefits are entirely negated by actual disposal pathways (43, 44). However, it is critical to recognize that the clinical viability of these novel materials for CAT remains underexplored. The dynamic intraoral environment presents severe challenges, including continuous thermal fluctuation, complex loading cycles, and exposure to saliva, that can rapidly compromise the biomechanical properties, fatigue resistance, and precise force delivery required for predictable tooth movement. Until rigorous clinical trials confirm their efficacy and safety, the adoption of biodegradable aligners in pre-prosthetic treatment planning cannot be recommended as a standard of care. Central to material/ instrument reuse is the established “4Rs” framework (Reduce, Reuse, Recycle, and Rethink), embodying circular economy principles that shift CAT away from its traditional linear “take-make-dispose” model toward a more regenerative, resource-efficient paradigm (45, 46). While some aligner manufacturers have initiated take-back programs, the large-scale feasibility of recycling used aligners remains limited. Used aligners are universally classified as contaminated medical waste due to biological exposure, necessitating specialized, energy-intensive sterilization protocols before any downstream processing. Furthermore, the multi-layered composition of many modern aligners, engineered specifically to balance rigidity and elasticity, complicates standard mechanical and chemical separation processes. Consequently, while these corporate recycling schemes represent a positive conceptual shift, they do not yet offer a validated, scalable solution for closed-loop material recovery in clinical practice.

While the 4Rs framework provides a valuable starting point for conceptualizing sustainable practice in dentistry, it is too broad to adequately guide clinical decision-making in prosthodontics. It focuses primarily on waste management and resource minimization without explicitly accounting for patient-related factors, prosthodontic biomechanics, complex multi-material systems, or the highly structured diagnostic-treatment-maintenance continuum that characterizes prosthodontic care. Additionally, its domains do not map cleanly onto the key environmental dimensions highlighted in this scoping review nor onto the sustainability drivers identified thematically. To address these limitations and bridge the gap between theoretical environmental goals and daily clinical decision-making, we propose the ProSustain-P (Practice Sustainability Pathway) framework. This specialty-specific model restructures sustainability into four interdependent domains aligned with the phases of prosthodontic care:

1. Patient & Planning (Plan): The planning domain encourages orthodontists to integrate patient factors, prognosis, and lifetime restoration pathways when selecting treatment options, including adjunctive clear aligner therapy, thereby balancing functional and esthetic objectives with long-term resource stewardship.
2. Procedures & Workflows (Perform): The procedural domain emphasizes optimization of clinical and laboratory workflows through appropriate digitization, appointment consolidation, and energy- and water-efficient operations to reduce waste and unnecessary remakes while maintaining prosthetic quality.
3. Materials & Devices (Provide): The materials and devices domain extends the logic of the 4Rs by prioritizing durable, repairable, and safer materials, designing restorations for longevity rather than replacement, and rationalizing the use and potential recycling of models, resins, and instruments. This domain emphasizes rationalizing the use of resins and models through digital workflows. It also encourages clinicians to critically evaluate manufacturer claims regarding recyclability, advocating instead for designs that minimize multi-material complexity where possible, thus improving the theoretical odds of future material recovery.
4. Practice & Planet Context (Proof & Progress): the practice and planet domain situates individual clinical choices within broader system responsibilities, encouraging implementation of sustainability metrics, staff and patient education, engagement with take-back or recycling schemes, and collaboration with industry and professional bodies.

The literature highlights the significance of chemical safety, driven by regulatory demands and health concerns over compounds such as bisphenol A and phthalates (47). Considering the prolonged intraoral wear of aligners, minimizing chemical leaching is essential to protect patients and limit environmental release of deleterious substances. While less frequently investigated, energy consumption and water utilization are progressively acknowledged as substantial determinants of CAT's environmental impact. Optimizing energy-intensive manufacturing processes, including thermoforming and specific 3D printing can be achieved through improved equipment, process re-engineering, and advanced digital workflows (48, 49).

Similarly, water consumption during model fabrication and appliance finishing can be reduced through direct printing and water-efficient clinical devices (50, 51). These observations resonate with broader global trajectories in sustainable dental materials research, emphasizing the criticality of a comprehensive, multifaceted methodology for minimizing the ecological burden of clear aligner therapy. Despite growing attention to materials and chemical safety, energy consumption and water use remain underexplored in sustainable CAT. The ProSustain-P framework directly addresses these overlooked areas by incorporating them into each domain, providing a comprehensive environmental strategy that aligns with the specialized needs of prosthodontic practice.

### Implications, limitations, and future directions

This scoping review carries significant implications for practitioners, researchers, industry, policymakers, and patients. Sustainable CAT aligns directly with the principle of minimally invasive dentistry. By utilizing clear aligners to pre-restoratively position teeth without invasive mechanical preparation, clinicians inherently reduce the consumption of prosthetic materials and the generation of associated laboratory waste over the patient's lifetime. Therefore, the most sustainable prosthetic outcome is often the one that preserves the most natural tooth structure through effective preventive regimes. Prevention of disease, being the single best route to mitigating environmental impacts.

The proposed “ProSustain-P” framework provides a practical guide, emphasizing optimized prosthodontic treatment planning to reduce aligner use, exploring reusable systems enabled by shape-memory polymers, establishing effective recycling programs, and holistically re-evaluating the aligner lifecycle to integrate esthetic, functional, and environmental considerations while delivering optimal patient care.

Despite progress, notable gaps remain. Most research consists of narrative reviews and theoretical papers, with few standardized measures of sustainability or comprehensive real-world studies. Methodological difficulties, limited resources, and inconsistent terminology continue to slow progress. There is an urgent need for more practical studies, especially RCTs comparing traditional and sustainable aligner materials in clinical settings. Long-term evaluation of environmental impacts across aligner systems would strengthen evidence-based practice. Moreover, exploring patient perspective and acceptance of sustainable restorative options could inform education and marketing strategies to increase demand for environmentally responsible care.

### Practice & policy roadmap for sustainable clear aligner practice

Guided by the evidence map, we synthesized a Practice & Policy Roadmap (Table 3) that aligns the four environmental dimensions with the ProSustain-P framework and assigns concrete actions to clinics (including practitioners and patients), manufacturers, and policy/education stakeholders. Each action is prioritized by feasibility and expected impact (plastic mass, energy/CO_2_e, water) and paired with practical, implementable metrics for clinical audit and performance tracking.

Practitioners: Esthetic dentistry specialists should integrate sustainable practices into treatment planning and daily operations. This involves selecting CAT from manufacturers with demonstrated environmental commitments, minimizing clinical waste, educating patients on proper disposal, and advocating for industry-wide standards. Cost savings show environmental responsibility can align with business efficiency.

Researchers: Comprehensive life cycle assessments are needed to quantify the environmental impacts of CAT from material extraction to disposal. Comparative studies should evaluate both environmental and clinical performance of new, biodegradable materials. Water usage in manufacturing and clinical settings also warrants deeper investigation, given its underrepresentation in current research.

Policymakers and professional organizations: Developing evidence-based guidelines and sustainability standards for clear aligners is critical. Regional research concentration underscores the need for international collaboration. Professional organizations can drive progress through certification programs, sustainability-focused education, and industry partnerships (18).

Manufacturers: Bio-based and biodegradable innovative materials, represent a challenge and a significant market opportunity. Companies adopting sustainable manufacturing, closed-loop recycling, and transparent reporting may gain competitive advantages as practitioners and patients become increasingly environmentally conscious.

Patients: Patients play role in advancing sustainability. They can contribute by engaging with and taking personal responsibility for their oral healthcare, as co-owners and co-managers of their wellbeing. Patient-led prevention of disease is key to personal wellbeing and environmental sustainability, as a positive un-unintended consequence. They must understand that used aligners are contaminated medical waste and cannot be disposed of via household recycling. Through targeted education from their practitioners, patients should be directed to return used aligners to the clinic for appropriate medical waste processing or manufacturer take-back programs. Ultimately, by prioritizing preventive care and adhering to proper waste protocols, patients actively contribute to a more sustainable dental practice.

## Conclusion

Sustainable CAT is an emerging field in esthetic dentistry, with evidence based on narrative and theoretical studies and limited standardization. Available limited evidence suggest that environmentally oriented strategies can reduce waste and chemical burden without compromising contemporary standards of esthetic care, but evidence on life-cycle impact, treatment outcomes under “environmentally sustainable” protocols, cost-effectiveness, and circular end-of-life pathways remain limited. The ProSustain-P framework distilled from this review provides a practical structure for clinicians, industry, and policymakers to begin integrating waste reduction, energy and water efficiency, safer materials, and prosthetic-specific innovation into clear aligner workflows while guiding future research toward standardized, outcome-linked sustainability metrics.

## Data Availability

The original contributions presented in the study are included in the article, further inquiries can be directed to the corresponding authors.

## References

[1] AlMogbel AM. Clear aligner therapy: up to date review article. J Orthod Sci. (2023) 12(1):37. 10.4103/jos.jos_30_23 37881665 PMC10597356

[2] Korkut B Unal T Murat N Özcan M. Effect of prerestorative short-term clear aligner therapy in restorative treatment planning. J Prosthet Dent. (2025) 133(2):455–63. 10.1016/j.prosdent.2023.02.024 37179153

[3] Tamer I Öztas E Marsan G. Orthodontic treatment with clear aligners and the scientific reality behind their marketing: a literature review. Turk J Orthod. (2019) 32(4):241–46. 10.5152/TurkJOrthod.2019.18083 32110470 PMC7018497

[4] Bichu YM Alwafi A Liu X Andrews J Ludwig B Bichu AY. Advances in orthodontic clear aligner materials. Bioact Mater. (2023) 22:384–403. 10.1016/j.bioactmat.2022.10.006 36311049 PMC9588987

[5] Chong H Peh J Weir T Meade MJ. Patient experiences with clear aligners: a scoping review. Eur J Orthod. (2025) 47(3):cjaf017. 10.1093/ejo/cjaf017 40237388 PMC12001029

[6] Zhang H Bi S Zhang X. Impact of clear aligners on gingivitis incidence and prevention strategies in adolescents and adults: a prospective observational study. BMC Oral Health. (2025) 25(1):75. 10.1186/s12903-025-05439-y 39819290 PMC11737181

[7] Inchingolo AD Dipalma G Ferrara I Viapiano F Netti A Ciocia AM. Clear aligners in the growing patient: a systematic review. Children. (2024) 11(4):385. 10.3390/children11040385 38671602 PMC11049164

[8] Eǧlenen MN Yavan MA. Has the COVID-19 pandemic affected orthodontists' interest in various orthodontic appliances? Turk J Orthod. (2023) 36(4):216–23. 10.4274/TurkJOrthod.2023.2022.124 38164005 PMC10763601

[9] Christensen L Luther F. Adults seeking orthodontic treatment: expectations, periodontal and TMD issues. Br Dent J. (2015) 218(3):111–7. 10.1038/sj.bdj.2015.46 25686427

[10] Hung M Zakeri G Su S Mohajeri A. Profile of orthodontic use across demographics. Dent J (Basel). (2023) 11(12):291. 10.3390/dj11120291 38132429 PMC10742803

[11] Pawar M Takale P. Clear Aligners Market by Material, Type, Age Group, Distribution Channel, End User — Global Forecast to 2030. Meticulous Research (2023). Available online at: https://www.meticulousresearch.com/product/clear-aligners-market-5331 (Accessed January 9, 2026).

[12] Kau CH Soh J Christou T Mangal A. Orthodontic aligners: current perspectives for the modern orthodontic office. Medicina (B Aires). (2023) 59(10):1773. 10.3390/medicina59101773 PMC10608554 37893491

[13] Macrì M D'Albis V Marciani R Nardella M Festa F. Towards sustainable orthodontics: environmental implications and strategies for clear aligner therapy. Materials (Basel). (2024) 17(17):4171. 10.3390/ma17174171 39274561 PMC11395928

[14] Khosravani MR Reinicke T. On the environmental impacts of 3D printing technology. Appl Mater Today. (2020) 20:100689. 10.1016/j.apmt.2020.100689

[15] Ferreira M Costa H Veiga N Correia MJ Gomes ATPC Lopes PC. Do clear aligners release toxic chemicals?—a systematic review. J Funct Biomater. (2025) 16(5):173. 10.3390/jfb16050173 40422837 PMC12112703

[16] Alkhamees A. The new additive era of orthodontics: 3D-printed aligners and shape memory polymers—the latest trend—and their environmental implications. J Orthod Sci. (2024) 13:55. 10.4103/jos.jos_211_23 39758107 PMC11698253

[17] Taymour N Alabdrubalameer DI Al Shooq ZH Al Yaseen MS Al Zaher RH Shetty AC. Dimensional accuracy of polyether elastomeric impression materials after using chitosan as a disinfectant: a sustainable approach to dental infection control. Prosthesis. (2025) 7(1):7. 10.3390/prosthesis7010007

[18] Eliades T Adel SM Akyalçin S Atsawasuwan P Foong KW Hiskia A. Environmental footprints in orthodontics: the world federation of orthodontists' white paper on sustainable practices, challenges and research imperatives. J World Fed Orthod. (2025) 14(4):194–201. 10.1016/j.ejwf.2025.06.003 40675890

[19] Shinkai RSA Biazevic MGH Michel-Crosato E de Campos TT. Environmental sustainability related to dental materials and procedures in prosthodontics: a critical review. J Prosthet Dent. (2025) 133(6):1466–73. 10.1016/j.prosdent.2023.05.024 37709614

[20] Robbins JW Alvarez MG Beckel BT Norris RT Caesar RR. Restoratively guided orthodontic treatment: the pre-orthodontic bonding concept. J Esthet Restor Dent. (2023) 35(1):270–8. 10.1111/jerd.12919 35575348

[21] O'Neill G. Porcelain rehabilitation of the maxillary arch following clear aligner orthodontic treatment. Aesthetic Update. (2024) 1(3):144–54. 10.12968/aedu.2024.1.3.144

[22] Weinstein T Marano G Aulakh R. Five-to-five clear aligner therapy: predictable orthodontic movement for general dentist to achieve minimally invasive dentistry. BMC Oral Health. (2021) 21(1):671. 10.1186/s12903-021-02034-9 34965879 PMC8717640

[23] Tai S. An interdisciplinary approach to restorative treatment with clear aligners. J Cosmetic Dent. (2020) 35(4):84–94.

[24] Barbhuiya S Das BB Adak D. A comprehensive review on integrating sustainable practices and circular economy principles in concrete industry. J Environ Manage. (2024) 370:122702. 10.1016/j.jenvman.2024.122702 39366229

[25] Pinaeva LG Noskov AS. Biodegradable biopolymers: real impact to environment pollution. Sci Total Environ. (2024) 947:174445. 10.1016/j.scitotenv.2024.174445 38981547

[26] Panayi N Cha JY Kim KB. 3D printed aligners: material science, workflow and clinical applications. Semin Orthod. (2023) 29(1):25–33. 10.1053/j.sodo.2022.12.007

[27] Narongdej P Hassanpour M Alterman N Rawlins-Buchanan F Barjasteh E. Advancements in clear aligner fabrication: a comprehensive review of direct-3D printing technologies. Polymers (Basel). (2024) 16(3):371. 10.3390/polym16030371 38337260 PMC10856925

[28] Arqub SA Voldman R Ahmida A Kuo CL Godoy LDC Nasrawi Y. Patients' perceptions of orthodontic treatment experiences during COVID-19: a cross-sectional study. Prog Orthod. (2021) 22(1):17. 10.1186/s40510-021-00363-7 34101037 PMC8185310

[29] Ruan C Xiong J Li Z Zhu Y Cai Q. Study on decision-making for orthodontic treatment plans based on analytic hierarchy process. BMC Oral Health. (2024) 24(1):488. 10.1186/s12903-024-04281-y 38658882 PMC11040963

[30] Olteanu ND Romanec C Cernei ER Karvelas N Nastri L Zetu IN. Scoping review—the effectiveness of clear aligners in the management of anterior open bite in adult patients. Medicina (B Aires). (2025) 61(6):1113. 10.3390/medicina61061113 PMC12195066 40572801

[31] Papadimitriou A Mousoulea S Gkantidis N Kloukos D. Clinical effectiveness of Invisalign® orthodontic treatment: a systematic review. Prog Orthod. (2018) 19(1):37. 10.1186/s40510-018-0235-z 30264270 PMC6160377

[32] Subbaraju K Goyal JD Patri V Panigrahi P Suthar MG Varma PK. Assessment of patient-reported outcomes in the use of clear aligners compared to conventional fixed braces. J Pharm Bioallied Sci. (2025) 17(Suppl 1):S457–9. 10.4103/jpbs.jpbs_1424_24 40511205 PMC12156765

[33] Martínez-Peláez R Escobar MA Félix VG Ostos R Parra-Michel J García V. Sustainable digital transformation for SMEs: a comprehensive framework for informed decision-making. Sustainability. (2024) 16(11):4447. 10.3390/su16114447

[34] Kumar S Smith SR Fowler G Velis C Kumar SJ Arya S. Challenges and opportunities associated with waste management in India. R Soc Open Sci. (2017) 4(3):160764. 10.1098/rsos.160764 28405362 PMC5383819

[35] Houssam N Ibrahiem DM Sucharita S El-Aasar KM Esily RR Sethi N. Assessing the role of green economy on sustainable development in developing countries. Heliyon. (2023) 9(6):e17306. 10.1016/j.heliyon.2023.e17306 37389081 PMC10300370

[36] Goswami M Goswami PJ Nautiyal S Prakash S. Challenges and actions to the environmental management of bio-medical waste during COVID-19 pandemic in India. Heliyon. (2021) 7(3):e06313. 10.1016/j.heliyon.2021.e06313 33748452 PMC7962757

[37] Tao Y Destek MA Pata UK Khan Z. Environmental regulations and carbon emissions: the role of renewable energy research and development expenditures. Sustainabiliy. (2023) 15(18):13345. 10.3390/su151813345

[39] Nunes AR Dale J. Global trends in sustainable healthcare research: a bibliometric analysis. Future Healthc J. (2025) 12(2):100251. 10.1016/j.fhj.2025.100251 40470003 PMC12133695

[38] Chua MT Chung LYE Ng EY Lim HXY Cheung NMT Lim CKW. Climate change and environmental sustainability in emergency medicine: a narrative review. Ann Transl Med. (2025) 13(3):31. 10.21037/atm-25-57 40689074 PMC12272795

[40] Kazemi MZ Elamer AA Theodosopoulos G Khatib SFA. Reinvigorating research on sustainability reporting in the construction industry: a systematic review and future research agenda. J Bus Res. (2023) 167:114145. 10.1016/j.jbusres.2023.114145

[41] Roslan NS Lee YY Ibrahim YS Anuar ST Yusof KMKK Lai LA. Detection of microplastics in human tissues and organs: a scoping review. J Glob Health. (2024) 14:04179. 10.7189/jogh.14.04179 39175335 PMC11342020

[42] Onur N Alan H Demirel H Köker AR. Digitalization and digital applications in waste recycling: an integrative review. Sustainability. (2024) 16(17):7379. 10.3390/su16177379

[43] Dadras Chomachayi M Jalali-arani A Beltrán FR de la Orden MU Martínez Urreaga J. Biodegradable nanocomposites developed from PLA/PCL blends and silk fibroin nanoparticles: study on the microstructure, thermal behavior, crystallinity and performance. J Polym Environ. (2020) 28(4):1252–64. 10.1007/s10924-020-01684-0

[44] Injorhor P Trongsatitkul T Wittayakun J Ruksakulpiwat C Ruksakulpiwat Y. Biodegradable polylactic acid-polyhydroxyalkanoate-based nanocomposites with bio-hydroxyapatite: preparation and characterization. Polymers (Basel). (2023) 15(5):1261. 10.3390/polym15051261 36904502 PMC10007227

[45] Hariyani D Hariyani P Mishra S Kumar Sharma M. Leveraging digital technologies for advancing circular economy practices and enhancing life cycle analysis: a systematic literature review. Waste Manag Bull. (2024) 2(3):69–83. 10.1016/j.wmb.2024.06.007

[46] Alves MN Seixas C Castro A Leitão A. Promoting the transition to a circular economy: a study about behaviour, attitudes, and knowledge by university students in Portugal. Sustainability. (2024) 16(1):343. 10.3390/su16010343

[47] Manzoor MF Tariq T Fatima B Sahar A Tariq F Munir S. An insight into bisphenol A, food exposure and its adverse effects on health: a review. Front Nutr. (2022) 9:1047827. 10.3389/fnut.2022.1047827 36407508 PMC9671506

[48] Campilho RDSG Barbosa FB. Innovations in manufacturing processes and systems for sustainable practices. Processes. (2025) 13(7):2315. 10.3390/pr13072315

[49] Fidan I Naikwadi V Alkunte S Mishra R Tantawi K. Energy efficiency in additive manufacturing. Condensed review. Technologies. (2024) 12(2):21. 10.3390/technologies12020021

[50] Nadagouda MN Ginn M Rastogi V. A review of 3D printing techniques for environmental applications. Curr Opin Chem Eng. (2020) 28:173–8. 10.1016/j.coche.2020.08.002 34327115 PMC8318092

[51] Norman A Mursyid QIBMA Chong CH Cheah KH Ramarad S Yap TC. Pioneering clean water solutions: how cutting-edge resin-based 3D printing is driving sustainable remediation. Sustain Mater Technol. (2025) 45:e01525. 10.1016/j.susmat.2025.e01525

[52] Mani S Manerikar R Goenka Y. Microplastics in orthodontics: a review. Guident. (2023) 16(11). https://guident.net/articles/orthodontics/MICROPLASTICS-IN-ORTHODONTICS:-A-REVIEW.html

[53] Kilaru K. A critique on materials for orthodontic aligners through sustainable manufacturing. E3S Web Conf. (2021) 309:01035. 10.1051/E3SCONF/202130901035

[54] Peter E JM Ani George S. Are clear aligners environment friendly? Am J Orthod Dentofac Orthop. (2022) 161(5):619–20. 10.1016/j.ajodo.2021.12.012 35016811

[55] Gupta S Ahluwalia R Gupta N Rana S. Aligners- their properties and disposal. J Pharm Negat Results. (2022) 13:186–8. 10.47750/PNR.2022.13.S06.027

[56] Caelli C Tamburrino F Brondi C Razionale AV Ballarino A Barone S. Sustainability in healthcare sector: the dental aligners case. Sustainability. (2023) 15(24):16757. 10.3390/SU152416757

[57] Da Tan TY Duane B Hussein A Samsonova A Sizun G Shakerdi L. Environmental sustainability of post-orthodontic dental retainers: a comparative life-cycle assessment of hawley and essix retainers. Eur J Orthod. (2024) 46(2):cjae012. 10.1093/EJO/CJAE012 38488436 PMC10941639

[58] Raj DR Sowmya J Verma S Raj GP Chitra P. Awareness regarding clear aligner disposal among orthodontists and general dentists: a cross-sectional survey-based study. J Indian Orthod Soc. (2024) 58(4):353–62. 10.1177/03015742241264372

[59] Leach E. Degradation of orthodontic clear aligners from outdoor weathering exposure. Theses & Dissertations [Internet]. (2024). Available online at: https://digitalcommons.unmc.edu/etd/879 (Accessed 2025 August 13).

[60] Lecocq G. Aligneurs: mise en perspective de la composition chimique, du recyclage, des propriétés mécaniques et des besoins thérapeutiques – partie 1. Orthod Fr. (2024) 95(2):169–75. 10.1684/ORTHODFR.2024.151 39106191

[61] Panayi N Papageorgiou SN Eliades G Eliades T. Microplastics and orthodontic aligners: the concerns arising from the modernization of practice through polymers and plastics. J World Fed Orthod. (2024) 13(6):259–64. 10.1016/J.EJWF.2024.10.001 39567342

[62] Akhtar N Tahir A Qadir A Masood R Gulzar Z Arshad M. Profusion of microplastics in dental healthcare units; morphological, polymer, and seasonal trends with hazardous consequences for humans. J Hazard Mater. (2024) 479:135563. 10.1016/J.JHAZMAT.2024.135563 39226689

[63] Veseli E Veseli K Behluli E Veseli E Veseli K Behluli E. The carbon emissions of clear aligner therapy: a critical review. APOS Trends in Orthod. (2024) 14(2):70–1. 10.25259/APOS_34_2024

[64] Ong A Teo JYQ Watts DC Silikas N Lim JYC Rosa V. The global burden of plastics in oral health: prospects for circularity, sustainable materials development and practice. RSC Sustainability. (2024) 2(4):881–902. 10.1039/D3SU00364G

[65] Palmieri E Montaina L Bellisario D Lucarini I Maita F Ielmini M. Towards green dentistry: evaluating the potential of 4D printing for sustainable orthodontic aligners with a reduced carbon footprint. Polymers (Basel). (2024) 16(24):3566. 10.3390/POLYM16243566 39771418 PMC11679438

[66] Choi W Mangal U Yu JH Ryu JH Kim J Jun T. Viscoelastic and antimicrobial dental care bioplastic with recyclable life cycle. Nat Commun. (2024) 15(1):1–14. 10.1038/s41467-024-53489-7 39448605 PMC11502779

[67] Peter E Monisha J Sylas VP George SA. How environmentally friendly is the disposal of clear aligners? A gas chromatography-mass spectrometry study. Am J Orthod Dentofac Orthop. (2025) 167(1):39–46. 10.1016/j.ajodo.2024.08.011 39352330

[68] Shrivastava P Ganiger C Pawar R Phaphe S Ronad Y Mane P. Eco-Conscious orthodontics: a greener approach to dental care. Cureus. (2025) 17(2):e78809. 10.7759/CUREUS.78809 40078266 PMC11897922

[69] Camcı H Büyükbayraktar ZÇ. Aligners from another perspective: could they be a long-term environmental threat? Problems and potential remedies. Am J Orthod Dentofac Orthop. (2025) 167(3):256–60. 10.1016/j.ajodo.2024.10.016 39641708

[70] Manochehri A Bhandi S Licardi FW Patil S. Sustainable Smiles: green orthodontics for a better tomorrow. annual research symposium [Internet]. (2025). Available from: Available online at: https://ecommons.roseman.edu/researchsymposium/2025/basic_sciences/9 (Accessed September 3, 2025).

[71] Gandhi R Veerasekaran A. An overview on environmental hazards of clear aligners. Int J Chem Biochem Sci. (2023) 23:367–70.

