# Supplementary material for: Sustainable clear aligner practice in esthetic and prosthetic dentistry: a scoping review for practical implementation
Source: Front Oral Health. 2026 Jul 2;7:1854402. doi: 10.3389/froh.2026.1854402 (PMC13373085; doi:10.3389/froh.2026.1854402)
Supplement: Supplementary file 1 [file Table1.docx]

**Supplemental file 1**

**Supplemental table 1: PICO framework and Search strategies**

| **PICO Framework**  Using a PICO framework, the Population (P) included all stakeholders involved in CAT —patients, clinicians/clinics, manufacturers, and the supply chain—across any clinical setting and geographic location. The Intervention (I) was any green or sustainable practice linked to CA throughout the product life cycle (e.g., material innovation, waste reduction/recycling/take-back programs, energy or water efficiency, safer chemical profiles, eco-conscious packaging/logistics, and education or policy initiatives). The Comparator (C) included conventional or non-sustainable practices, alternative workflows/appliances, or none where studies were descriptive (e.g., LCA or modeling). Outcomes (O) required at least one measure related to environmental impact (e.g., life-cycle indicators, emissions, microplastics/toxicity), resource use (materials, energy, water), or economic/operational implications (costs, efficiency, feasibility/adoption). |
| --- |
| **Search strategies**  The PubMed search strategy included MeSH terms and keywords:  ("Clear Aligners"[Mesh] OR "Aligners" OR "Invisalign" OR "Thermoplastic aligners" OR "Plastisc" OR "Microplastic" OR "Removable orthodontic appliances") AND ("Sustainability"[Mesh] OR "Green Dentistry" OR "Green Orthodontics" OR "Eco-friendly" OR "Sustainable Materials" OR "Carbon Footprint" OR "Waste Management" OR "Recycling" OR "Biodegradable" OR "Plastic" OR "Microplastic" OR "Environmental Impact") AND ("Orthodontics"[Mesh] OR "Dental Practice" OR "Dentistry") |
| The Web of Science query was:  TS=("clear aligners" OR "Invisalign" OR "thermoplastic aligners" OR "removable orthodontic appliances") AND TS=("sustainability" OR "green dentistry" OR "eco-friendly" OR "carbon footprint" OR "waste management" OR "recycling" OR "biodegradable" OR "environmental impact" OR "sustainable materials" OR "life cycle assessment") AND TS=("orthodontics" OR "dental practice" OR "dentistry" OR "dental materials") |
| The Google Scholar query was:  "Clear Aligners" OR Aligners OR Invisalign OR "Thermoplastic Aligners" OR Plastic OR Microplastic OR "Removable Orthodontic Appliances" AND Sustainability OR "Green Dentistry" OR "Green Orthodontics" OR "Eco-friendly" OR "Sustainable Materials" OR "Carbon Footprint" OR "Waste Management" OR Recycling OR Biodegradable OR "Environmental Impact" AND Orthodontics OR "Dental Practice" OR Dentistry” |
